# Supplementary material for: Some CSF Kynurenine Pathway Intermediates Associated with Disease Evolution in Amyotrophic Lateral Sclerosis
Source: Biomolecules. 2021 May 5;11(5):691. doi: 10.3390/biom11050691 (PMC8147980; doi:10.3390/biom11050691)
Supplement: Supplementary file 1 [file biomolecules-11-00691-s001.zip › biomolecules-1200674-supplementary.pdf]

a)

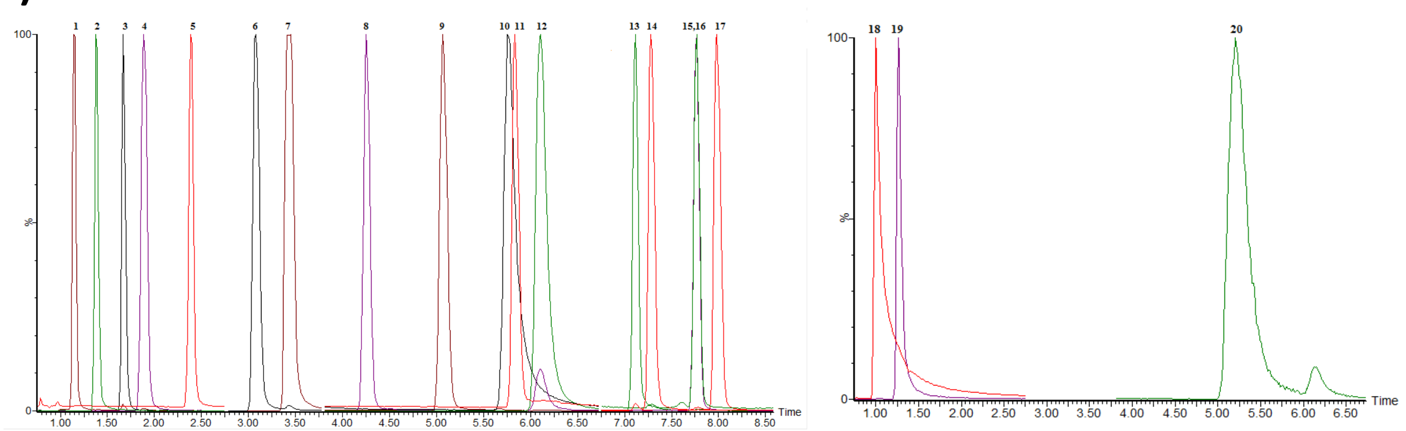

b)

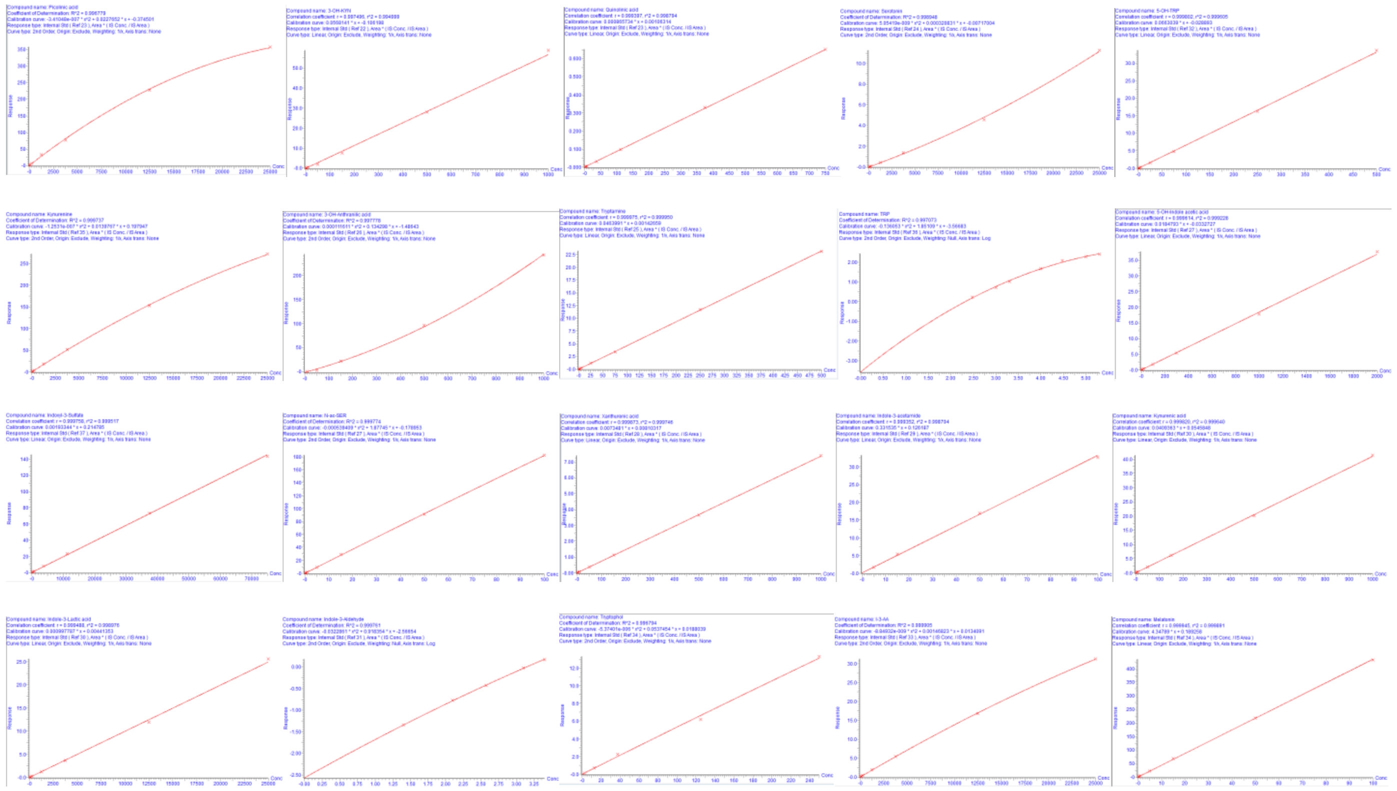

Supplementary Figure 1: Example of chromatograms and calibration curves for the determination of Kynurenine pathway intermediates. (a) Example of chromatograms : 1, 3-OH kynurenine; 2, Serotonin; 3, 5OH-tryptophan; 4, Kynurenine; 5, 3OH-anthranilic acid; 6, Tryptamine; 7, Tryptophan; 8, 5-HIAA; 9, N-acetylserotonin; 10, Xanthurenic acid; 11, Indole-3-acetamide; 12, Kynurenic acid; 13, Indole-3-lactic acid; 14, Indole-3-aldehyde; 15, Tryptophol; 16, Indole-3-acetic acid; 17, Melatonin; 18, Picolinic acid; 19, Quinolinic acid; 20, Indoxyl-3-sulfate. **(b)** Calibration curves used to determine the concentrations of each metabolite in patient samples

Supplementary table 1 : Concentrations, retention time, internal standards et ionization used for determination of tryptophan metabolism intermediates.

Range Concentrations (pmol/mL)	Retention time (min)	Metabolites	Internal standard	Concentrations IS (pmol/mL)	Ionisation	Dwell time (s)	Cone voltage (V)
37.5 - 25000	1,01	Picolinic acid	Picolinic-d3 Acid	2000	Positive	0,044	20
1.5 - 1000	1,15	3-OH-kynurenine	3-Hydroxy Kynurenine-$^{13}C_2$,^{15}N	20	Positive	0,044	5
1.13 - 750	1,26	Quinolinic acid	QUIN-d3	250	Negative	0,044	10
37.5 - 25000	1,39	Serotonin	Serotonin-d4	2000	Positive	0,044	10
0.75 - 500	1,68	5-Hydroxytryptophane	5-Hydroxy L-Tryptophan-d4	40	Positive	0,044	10
37.5 - 25000	1,87	Kynurenine	L-Kynurenine-d4	50	Positive	0,044	10
1.5 - 1000	2,41	3-OH-anthranilic acid	3-Hydroxyanthranilic Acid-d3	50	Positive	0,044	10
0.75 - 500	3,01	Tryptamine	Tryptamine-d4	20	Positive	0,197	30
300 - 200000	3,36	Tryptophan	L-Tryptophan-d5	2000	Positive	0,197	5
3 - 2000	4,23	5-Hydroxyindole acetic acid	5-Hydroxyindole-3-acetic acid-d5	75	Positive	0,066	10
112.5 - 75000	4,92	Indoxyl-3-Sulfate	I-3-Sulfate $^{13}C_6$	1000	Negative	0,066	10
0.15 - 100	5,05	N-acetyserotonine	Xanthurenic Acid-d4	20	Positive	0,066	5
1.5 - 1000	5,67	Xanthurenic acid	Xanthurenic Acid-d4	20	Positive	0,066	10
0.15 - 100	5,79	Indole-3-acetamide	1H-Indole-d5-3-acetamide	40	Positive	0,066	19
1.5 - 1000	6,04	Kynurenic acid	Kynurenic Acid-d5	20	Positive	0,066	10
37.5 - 25000	7,05	Indole-3-lactic acid	Kynurenic Acid-d5	20	Negative	0,066	5
3.75 - 2500	7,25	Indole-3-aldehyde	I-ALD-^{13}C	500	Positive	0,066	38
0.38 - 250	7,70	Tryptophol	Indoleacetic Acid-d4	1000	Positive	0,066	0
37.5 - 25000	7,70	Indole-3-acetic acid	Indoleacetic Acid-d4	1000	Positive	0,066	10
0.15 - 100	7,94	Melatonin	Melatonin-d4	2	Positive	0,066	38

Supplementary table 2 : mobile phase gradient used for determination of tryptophan metabolism intermediates

Temps (min)	%A	%B
0	95	5
2	90	10
4	85	15
6	70	30
8	60	40
9	55	45
9.5	0	100
11	0	100
11.5	95	5
13.5	95	5

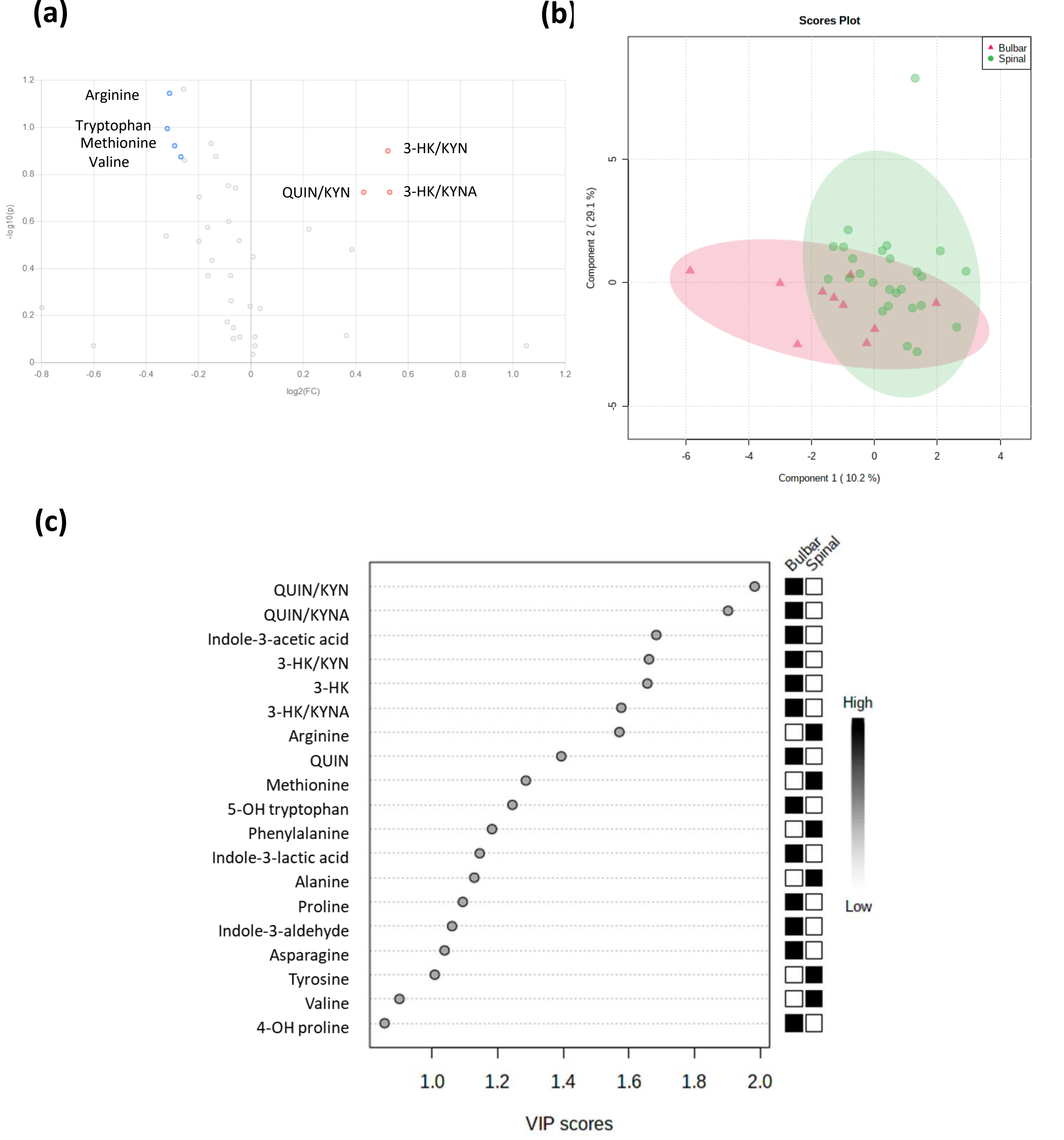

Supplementary Figure 2: Multivariate Analysis of CSF amino acids and tryptophan catabolism metabolites between bulbar and spinal onset. (a) Volcano plot representing the most important features in univariate analysis. Blue dots indicate a decrease of the feature in patients who had a bulbar onset and red dots an increase. **(b)** Scores plots of the PLS-DA model. Red triangles represent ALS patients with a bulbar onset and green dots represents patients who had a spinal onset, **(c)** Important features (VIP > 0.8) identified by PLS-DA, The boxes on the right indicate the relative concentrations of the corresponding metabolite in each group

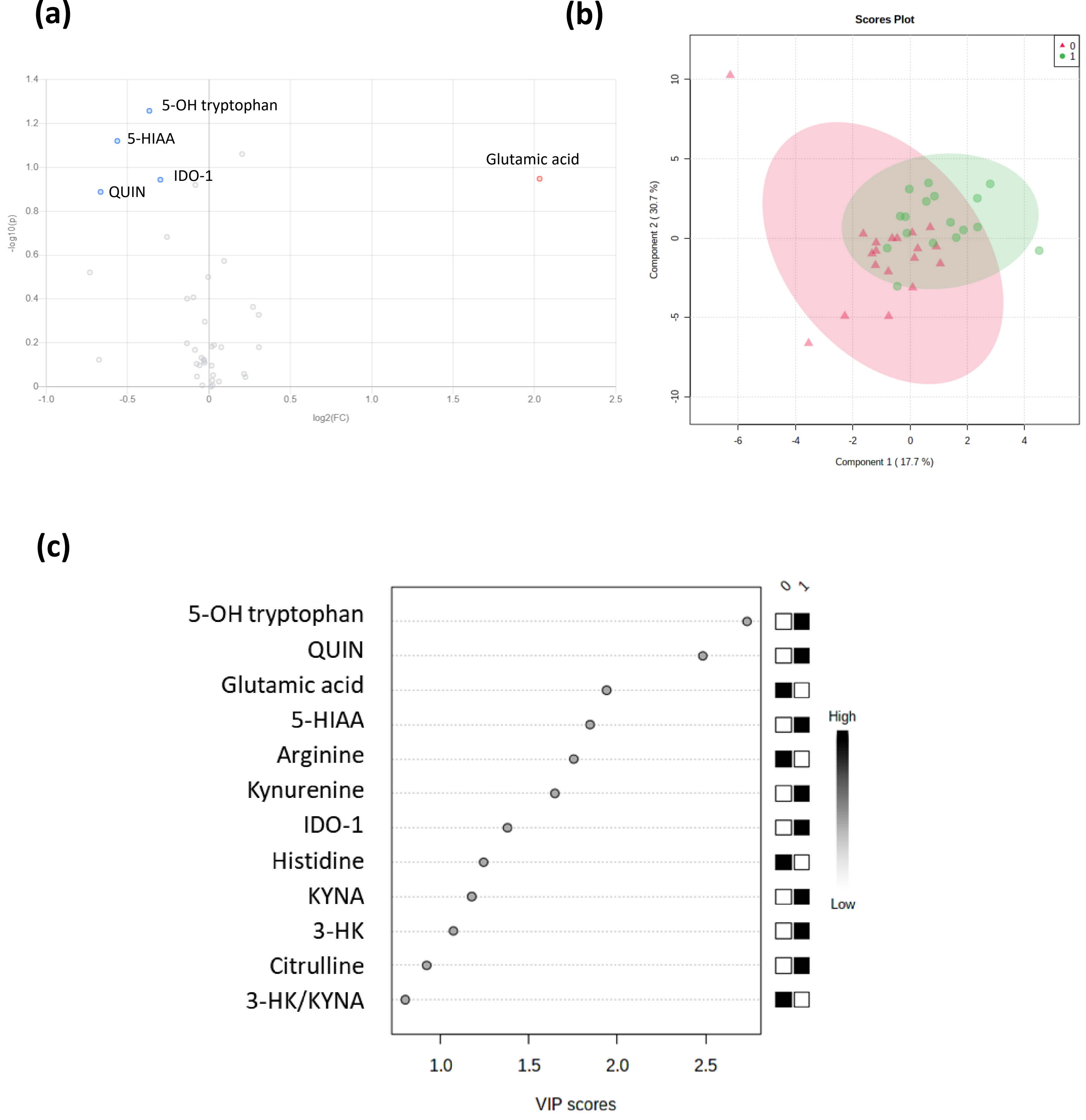

Supplementary Figure 3: Multivariate Analysis of CSF amino acids and tryptophan catabolism metabolites for the age at the first symptoms . Two groups have been constructed according to the median (67 years), **(a)** Volcano plot representing the most important features in univariate analysis. Blue dots mean a decrease of the feature in younger patients, red dots means an increase, (**b)** Scores plots of the PLS-DA model. Red triangles (0) represent younger ALS patients and green dots (1) represents older patients, **(c)** Important features (VIP > 0.8) identified by PLS-DA, The boxes on the right indicate the relative concentrations of the corresponding metabolite in each group

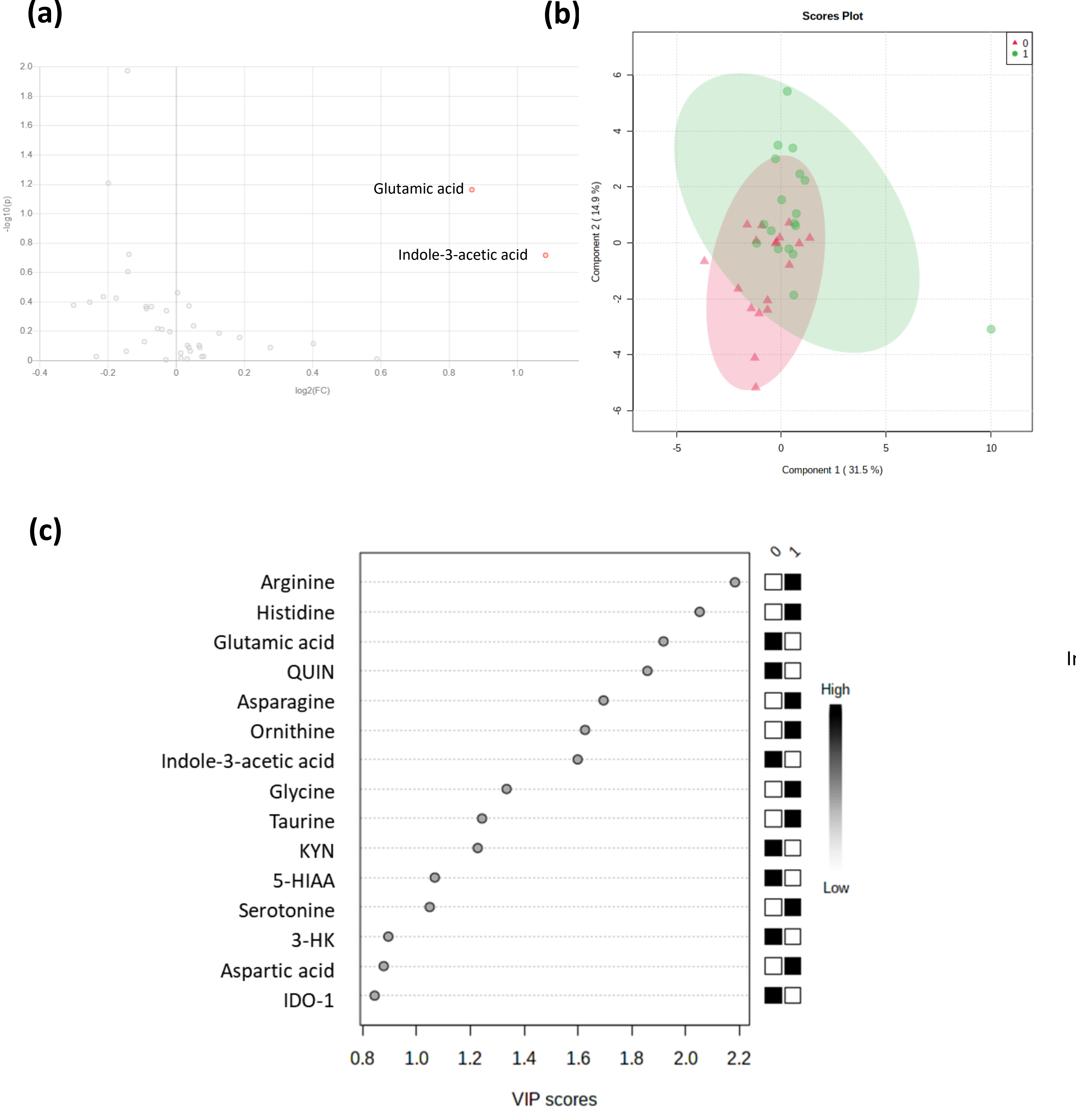

Supplementary Figure 4: Multivariate Analysis of CSF amino acids and tryptophan catabolism metabolites for the loss of weight at diagnostic. Two groups have been constructed according to the median variation (1.78 %), **(a)** Volcano plot representing the most important features in univariate analysis. Red dots mean an increase of the feature in patients who lost more than 1.78 % of their reference weight, **(b)** Scores plots of the PLS-DA model. Red triangles (0) represent ALS patients who lost more than 1.78 % of their weight reference and green dots (1) represents patients who lost less than 1.78 % of their weight reference, **(c)** Important features (VIP > 0.8) identified by PLS-DA, The boxes on the right indicate the relative concentrations of the corresponding metabolite in each group

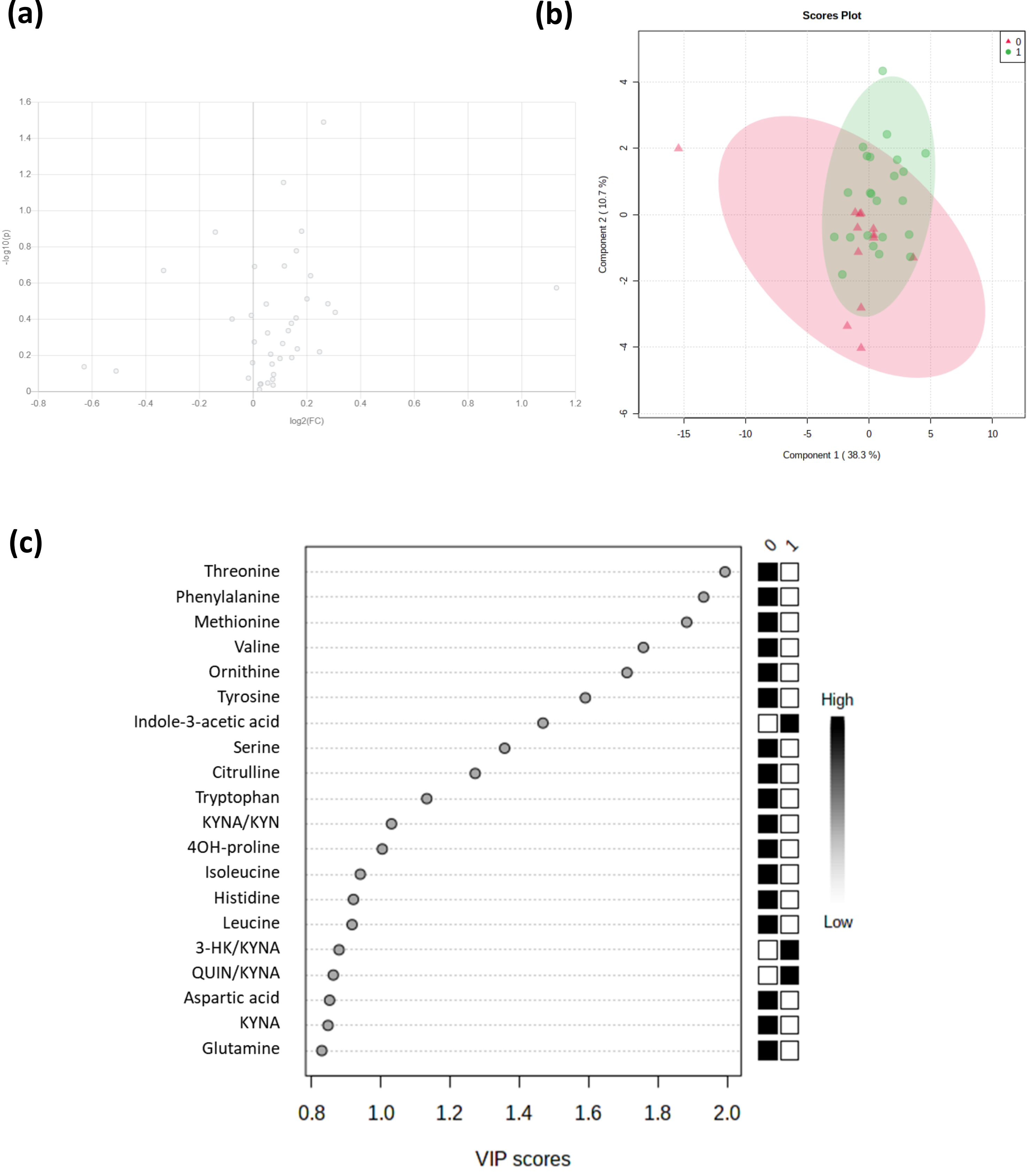

Supplementary Fig.5 Multivariate Analysis of CSF amino acids and tryptophan catabolism metabolites for the ALSFRS-r score at diagnostic. Two groups have been constructed according to the median (40), **(a)** Volcano plot representing the most important features in univariate analysis. No features were highlighted between patients with ALSFRS-r score at diagnostic less or superior than 40. **(b)** Scores plots of the PLS-DA model. Red triangles (0) represent ALS patients who had a ALSFRS-r score at diagnostic less than 40 and green dots (1) represents the others. **(c)** Important features (VIP > 0.8) identified by PLS-DA, The boxes on the right indicate the relative concentrations of the corresponding metabolite in each group

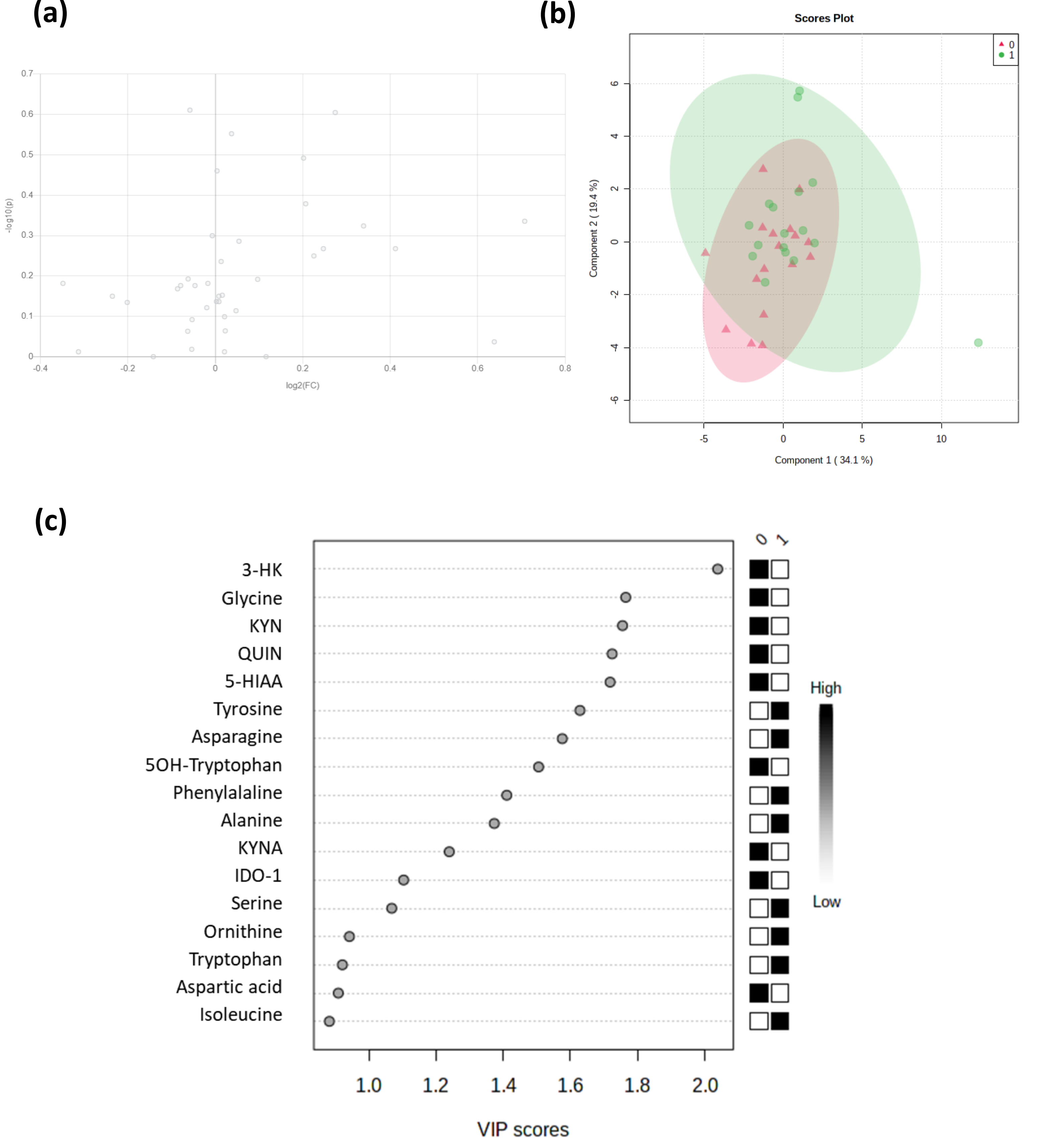

Supplementary Figure 6: Multivariate Analysis of CSF amino acids and tryptophan catabolism metabolites for the FVC score at diagnostic. Two groups have been constructed according to the median (40), **(a)** Volcano plot representing the most important features in univariate analysis. No features were highlighted between patients who had a FVC at diagnostic less or superior than 96.29% , red dots indicate an increase, **(b)** Scores plots of the PLS-DA model. Red triangles (0) represent ALS patients who had a FVC at diagnostic less than 96,29% and green dots (1) represents the others. **(c)** Important features (VIP > 0.8) identified by PLS-DA, The boxes on the right indicate the relative concentrations of the corresponding metabolite in each group

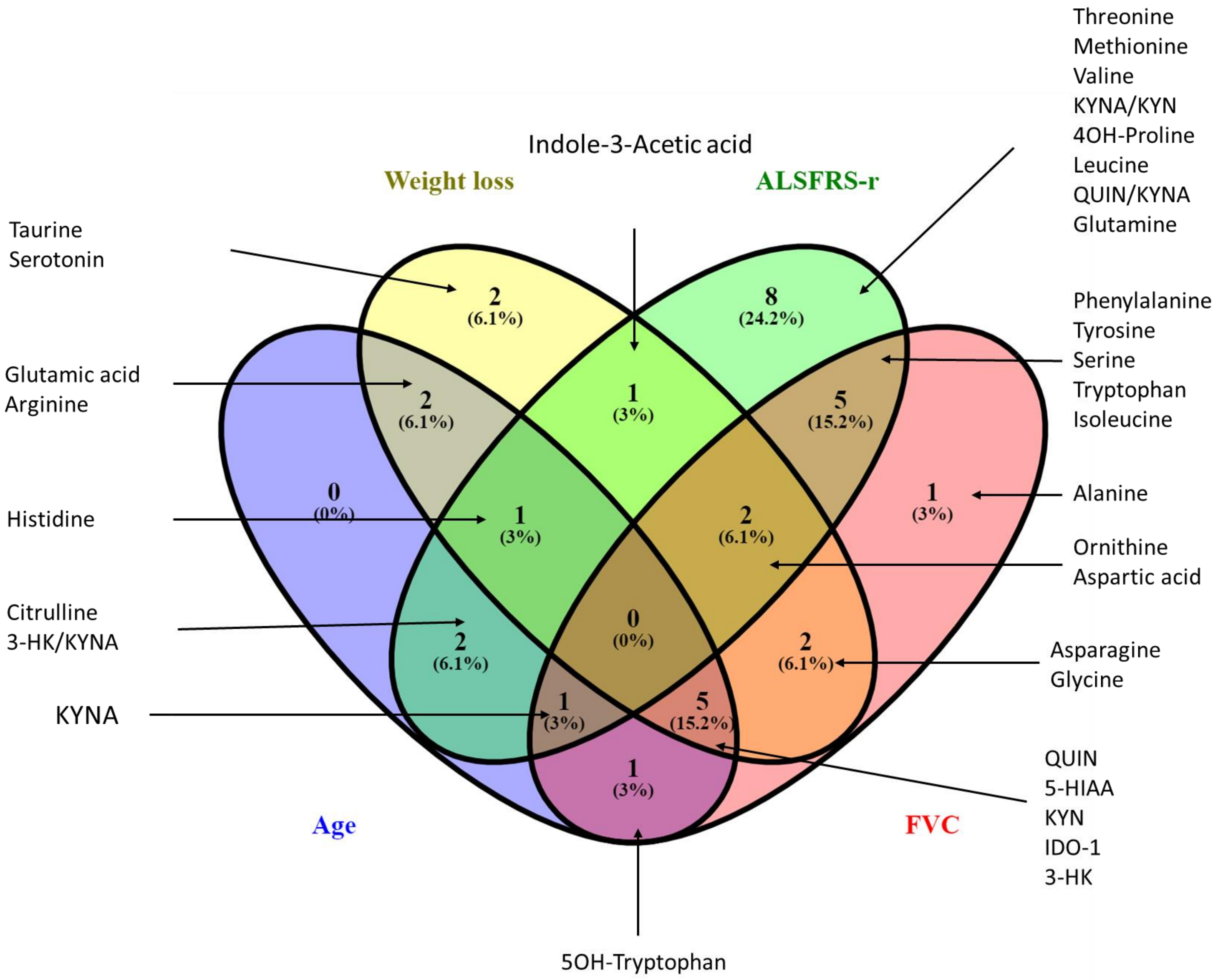

Supplementary Figure 7 : Venn diagram representing metabolites with a VIP > 0.8 with PLS-DA models between ALS patients according to age at first symptoms variation of Weight, FVC and ALSFRS-r score at diagnosis.

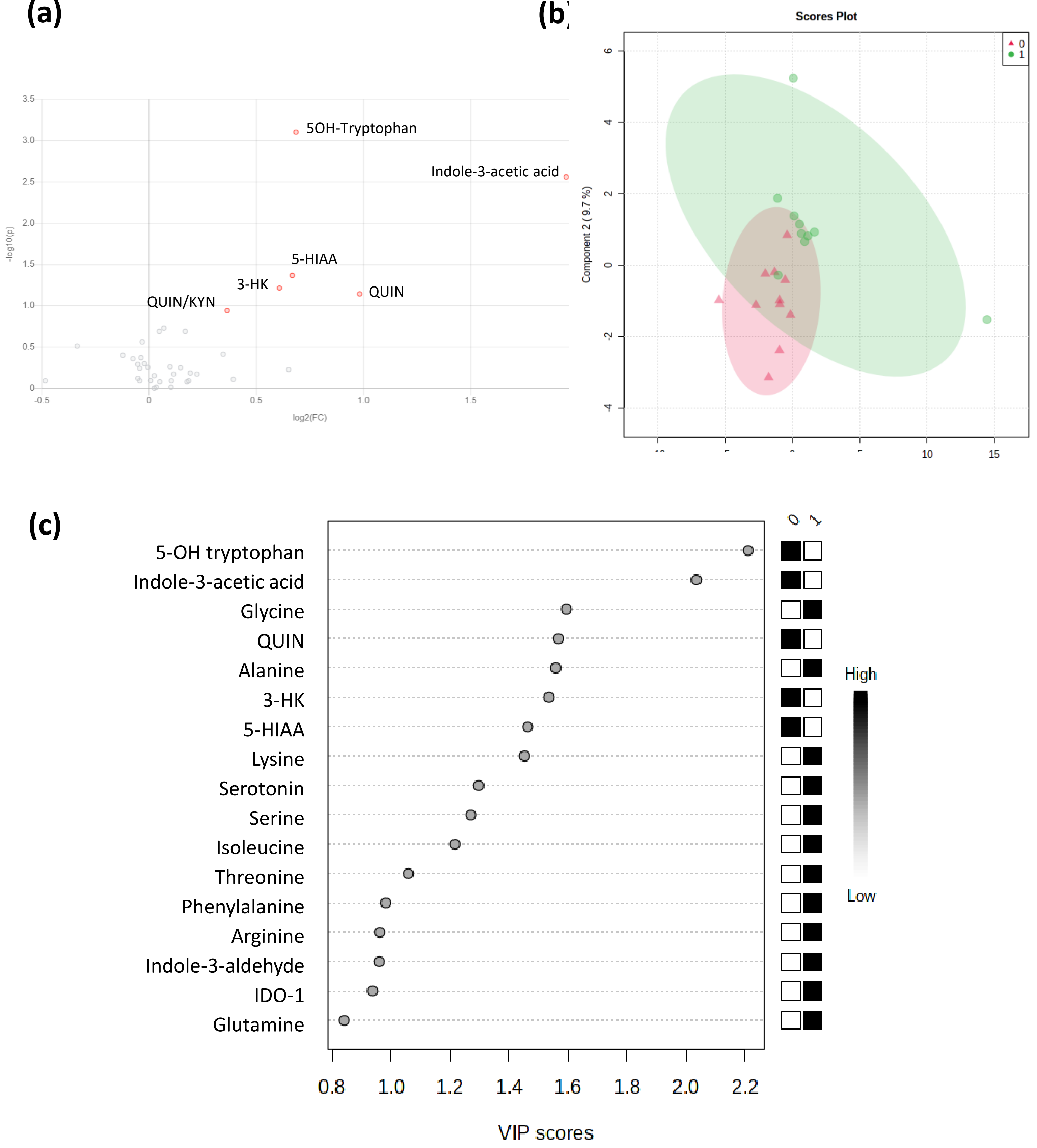

Supplementary Figure 8 : Multivariate Analysis of CSF amino acids and tryptophan catabolism metabolites for the weight loss at 12 months. Two groups have been constructed according to the median variation (1,49 %)**. (a)** Volcano plot representing the most important features in univariate analysis. Red dots indicate an increase of the feature in patients who had a loss of weight > 1,49% at 12 month compared to at diagnosis. **(b)** Scores plots of the PLS-DA model. Red triangles (0) represent ASL patients who have a loss of weight > 1,49% at 12 months compared to at diagnosis and green dots (1) represents patients who have a loss of weight < 1,49% at 12 months compared to at diagnosis or did not loos weight. **(c)** Important features (VIP > 0.8) identified by PLS-DA, The boxes on the right indicate the relative concentrations of the corresponding metabolite in each group

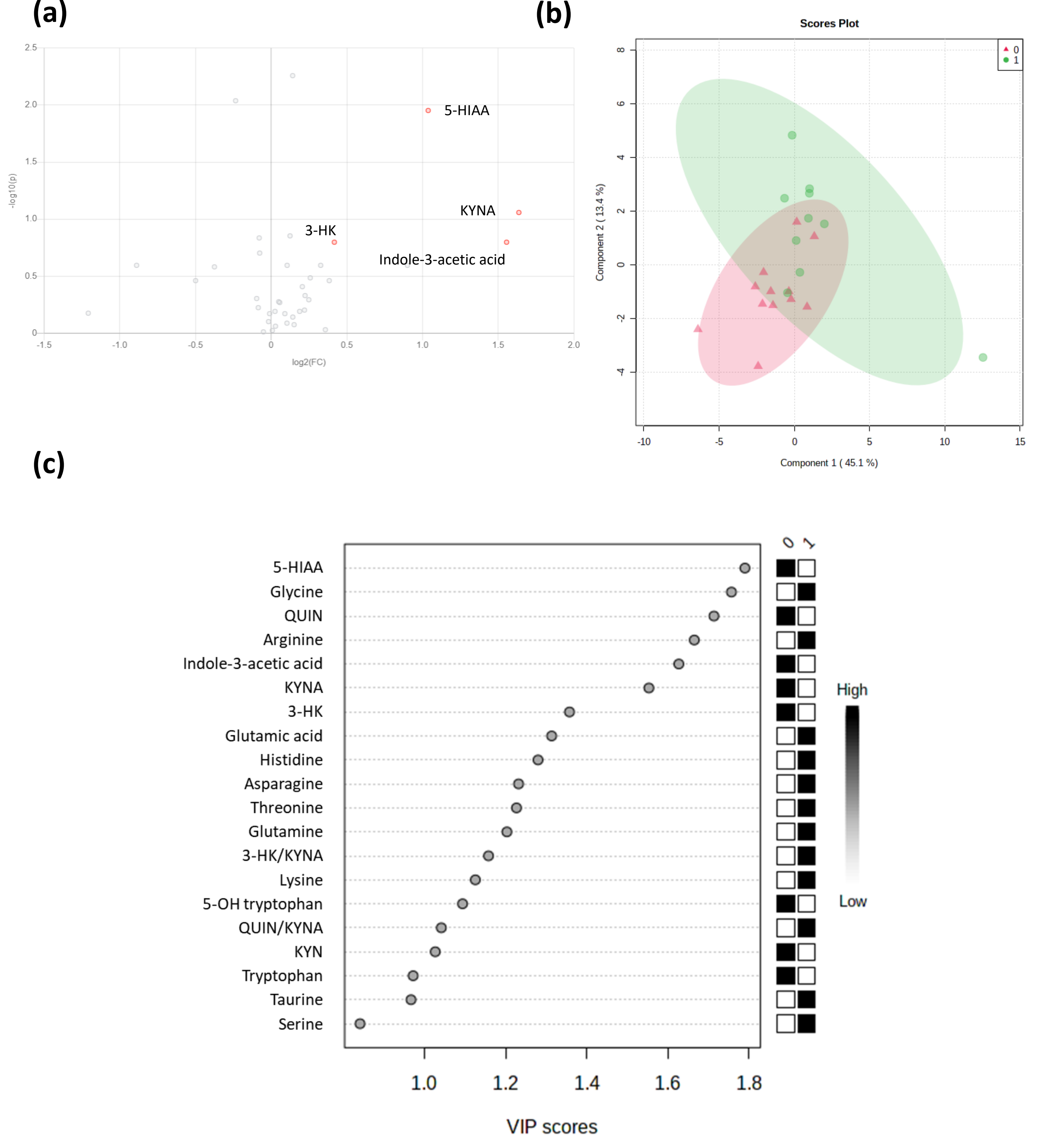

Supplementary Figure 9: Multivariate Analysis of CSF amino acids and tryptophan catabolism metabolites for ALSFRS-r score variant at 12 months.. Two groups were constructed according to the median variation (32%). **(a)** Volcano plot representing the most important features in univariate analysis. Red dots indicate an increase of the feature in patients who had a variation of ALSFRS-r score > 32%. **(b)** Scores plots of the PLS-DA model. Red triangles (0) represent ASL patients who experienced a reduction of ALSFRS-r score > 32% between diagnosis and 12 months later and green dots (1) represents patients who experienced a reduction of ALS score < 32%. **(c)** Important features (VIP > 0.8) identified by PLS-DA, The boxes on the right indicate the relative concentrations of the corresponding metabolite in each group

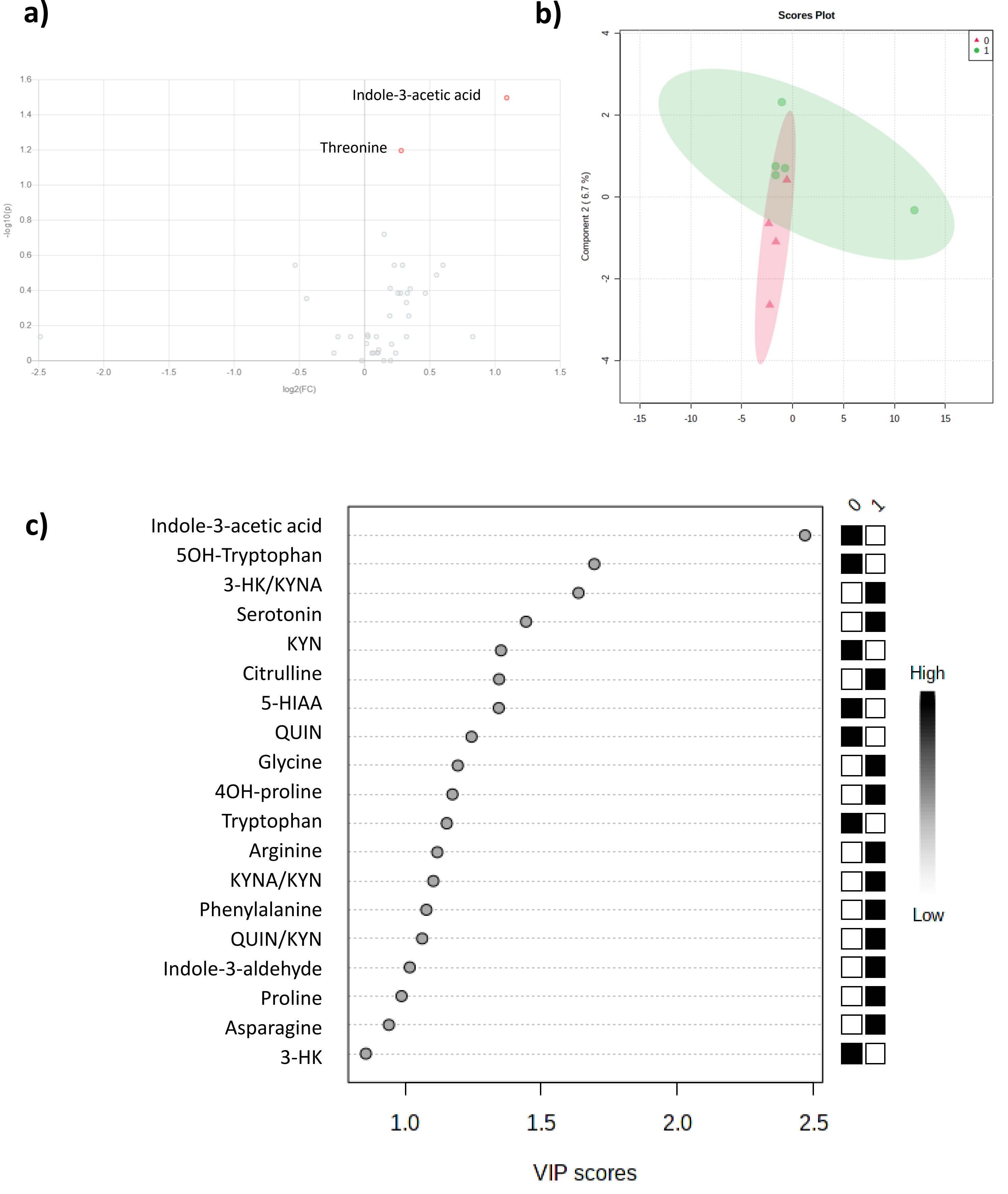

Supplementary Figure 10: Multivariate Analysis of CSF amino acids and tryptophan catabolism metabolites for FVC variation at 12 months. Two groups have been constructed according to the median variation (17.8 %). **(a)** Volcano plot representing the most important features in univariate analysis. Red dots indicate an increase of the feature in patients who had a reduction of FVC > 17.8% between the diagnostic and 12 months later. **(b)** Scores plots of the PLS-DA model. Red triangles (0) represent ASL patients who have a reduction of FVC > 17.8% between the diagnostic and 12 months later and green dots (1) represents patients who have a reduction of FVC < 17.8% between the diagnostic and 12 months later. **(c)** Important features (VIP > 0.8) identified by PLS-DA, The coloured boxes on the right indicate the relative concentrations of the corresponding metabolite in each group
